# Dynamic Role of Omega-3/Omega-6 Polyunsaturated Fatty Acid Ratio in Modulation of Adipogenicity, Lipid Metabolites, and Adipokines Associated with Platelet Hyperactivity

**DOI:** 10.3390/metabo16040271

**Published:** 2026-04-17

**Authors:** Sultanah Turki Almolafikh, Pandurangan Subash-Babu, Tlili Barhoumi, Ali A Alshatwi

**Affiliations:** 1 Department of Food Science and Nutrition, College of Food Science and Agriculture, King Saud University, Riyadh 11451, Saudi Arabia; 443204025@student.ksu.edu.sa (S.T.A.); subash@ksu.edu.sa (P.S.-B.); 2 Medical Research Core Facility and Platform (MRCFP), King Abdullah International Medical Research Center (KAIMRC), King Saud bin Abdulaziz University for Health Sciences (KSAU-HS), King Abdulaziz Medical City (KAMC), Ministry of National Guard Health Affairs (MNGHA), Riyadh 11426, Saudi Arabia; barhoumit@kaimrc.edu.sa

**Keywords:** essential fatty acid, adipose tissue, metabolic inflammation, prostaglandin and thromboxane

## Abstract

**Background:** Unhealthy expansion of adipose tissue (AT) due to excessive dietary intake of omega-6 or overnutrition stimulates the overaccumulation of the extracellular matrix (ECM), resulting in AT metabolic dysregulation. Hypertrophic conditions, excessive adipose depots, and hypoxia stimulate the overproduction of collagenous and non-collagenous proteins, which pathophysiologically initiate the pro-fibrotic signaling pathway associated with fibrosis progression, resulting in atherosclerosis and cardiovascular diseases. **Methods:** We aimed to investigate adipocyte plasticity in response to a varying ratio of omega-3 (ω3) to omega-6 (ω6) supplementation during the chemically induced adipogenic differentiation of human mesenchymal stem cells. Additionally, changes in lipid accumulation, adipocyte hypertrophy and hyperplasia, active lipid metabolites, and inflammatory cytokine profiles were evaluated. Furthermore, conditioned media from adipocytes treated with different ω3/ω6 ratios were applied to platelets to assess inflammatory responses through prostaglandin and thromboxane measurements. **Results:** A 1:3 ratio of ω3/ω6 (20:60 µM) significantly reduced lipid accumulation, promoted brown-like adipocyte morphology, and decreased apoptosis and reactive oxygen species (ROS) generation, as confirmed via FACS analysis. Transcriptional control of adipose tissue expansion was confirmed by the downregulation of LIPIN1 and COL1A1 mRNA expression and *p*-prostaglandin12-R protein levels in a 1:3 ratio when compared with 1:1, 1:2, 1:4, or 2:6 ratios of ω3/ω6. Notably, a 1:3 ratio of fatty-acid-treated adipocyte-conditioned media-treated platelets significantly reduced platelet activation and aggregation, as evidenced by lower *p*-thromboxane A2 protein levels. **Conclusions:** Supplementation with a 1:3 (20:60 µM) ω3/ω6 ratio favored the development of lean adipocytes, evidenced by the decreased lipid storage achieved by mitochondrial thermogenesis, which attenuated minimal adipocyte expansion and metabolic inflammation.

## 1. Introduction

In adipose tissue, metabolic homeostasis is predominantly maintained by white adipose tissue, which is recognized as an active endocrine organ [1]. Alterations in adipose tissue behavior play a crucial role in the progression of obesity-associated metabolic complications, such as insulin resistance, pro-thrombosis, and cardiovascular diseases [2]. Dietary intake of essential omega-3 (ω3) and omega-6 (ω6) fatty acids in a balanced proportion plays a dynamic role in the physiological functions and molecular actions of the synthesis of adipocyte cell membrane phospholipids, bioactive lipid metabolites, and particularly sphingolipids and phospholipids through cyclooxygenase and lipoxygenase pathways [3]. Polyunsaturated fatty acids and bioactive lipid mediators from essential ω6 fatty acids, such as arachidonic acid, prostaglandins, leukotrienes, and thromboxanes, are the key precursors for the biosynthesis of inflammatory mediators. Esterified arachidonic acid is mostly found in the mammalian cell membrane, but unesterified free arachidonic acid can act as a precursor for the biosynthesis of pro-inflammatory eicosanoids, which are mediated by various immune cells (macrophages and lymphocytes) [4,5]. Meanwhile, bioactive lipids synthesized from other ω3 fatty acids, such as docosahexaenoic acid (DHA) and eicosapentaenoic acid (EPA), are linked to anti-inflammatory characteristics with a critical role in adipose tissue inflammatory resilience via the inhibition of arachidonic acid and eicosanoid biosynthesis [5]. Dietary deficiency or imbalance between ω3 and excessive ω6 fatty acid develops adipose tissue inflammation, and alterations in adipocyte membrane lipoprotein synthesis or lipid accumulation lead to obesity-associated fatty liver diseases, defective adiponectin and collagen synthesis, fibrosis in adipocytes [6], and alterations in membrane phospholipids and immune cells such as leucocytes or platelet-stimulated low-grade inflammatory cytokines associated with pro-thrombosis [7].

Altered fatty acid bioactive metabolites have been proposed as a crucial factor for defective adipose tissue (AT) in lipid metabolite composition, followed by extracellular matrix (ECM) remodeling, which initiates the progression of obesity-associated metabolic inflammation, anti-atherogenesis, or low-grade chronic inflammation [8]. Metabolic inflammation arises during the shift between food restriction and feed-state rate-limiting enzymes, such as insulin-sensitive lipoprotein lipase (LPL), hormone-sensitive lipase (HSL), and monoacylglycerol lipase (MAGL) [9]. Surplus intake of ω6 arachidonic acid fatty acid involves the expansion of white adipocytes by up to 10-fold in diameter to store the excess fat as TAG, which leads to increased hypoxia and chronic inflammation [10]. During obesity, the expansion of WAT or hypertrophy (enlarged adipocyte size) and hyperplasia have a high collagen demand for ECM maintenance, and excessive demand restricts high-molecular-weight adiponectin formation, disrupts insulin receptor signaling, and stimulates the excessive synthesis of collagen from platelets via tissue factor-VI activation [11]. Collectively, this promotes fibrosis in WAT, resulting in adipocyte dysfunction, insulin resistance, and inflammation [12].

Under adipocyte hypertrophic/hyperplasia conditions, AT’s ECM proteins and proteases mediate ECM remodeling. This is an inevitable process that is pathologically accelerated in the high-fat-fed obese state [13]. Unusual expression of ECM components and fragments derived from tissue remodeling processes can impact immune cell recruitment and activation and may cause aggressive metabolic inflammation. ECM turnover requires a tightly regulated balance between the synthesis of the components and their proteolysis, mainly by fibrinolytic systems and matrix metalloproteases (MMPs) [14]. The compositions of ω3 and ω6 in red blood cell membranes represent a causative biomarker for prolonged dietary intake plus endogenous metabolism to predict weight gain and obesity progression [15]. Obesity progression is inversely associated with ω3-fatty-acid-rich RBC membrane phospholipids, while an excessive cis-ω6 fatty acid and ω3-to-ω6 ratio is positively associated with longitudinal weight gain. To our knowledge, there is no solid evidence of a favorable ratio of ω3/ω6 regulating adipocyte plasticity, AT homeostasis, and the inverse relationship with metabolic inflammation or crosstalk between platelets. The present study aimed to evaluate adipocyte intracellular lipid uptake, metabolic homeostasis, and inflammatory responses against different ratios of ω3/ω6 levels, such as 20:20, 20:40, 20:60, 20:80, and 40:60, during adipocyte plasticity and maturation upon differentiation from human mesenchymal stem cells. In addition, the conditioned media from adipocytes treated with different ratios of ω3/ω6 were examined to stimulate fibrosis formation in platelets. A detailed molecular mechanistic approach to explore adipocyte plasticity, ECM remodeling, and the progression of adipokines with different ratios of ω3/ω6 supplementation in the restricted environment of adipocyte differentiation is expected to facilitate the development of therapeutic approaches to control pro-thrombotic and atherosclerosis development.

## 2. Materials and Methods

### 2.1. Cell Lines, Cell Culture Reagents, and Chemicals

Bone marrow-derived human mesenchymal stem cells (PCS-500-012) were obtained from the American Type Culture Collection (ATCC, Manassas, VA, USA). In addition, human megakaryocytes (MEG-01 cells) were obtained from Cytion (Cytion™ Number 300482), Heidelberg, Germany. Cell culture materials, including Dulbecco’s modified Eagle’s medium (DMEM), Roswell Park Memorial Institute (RPMI 1640) medium, fetal bovine serum (FBS), trypsin, EDTA, and other materials, were purchased from Gibco (Paisley, UK). The antibiotics penicillin and streptomycin were purchased from Hyclone Laboratories (Logan, Utah, USA). Omega-3 (ω3) fatty acid, specifically *cis*, *cis*, *cis*-9,12,15-octadecatrienoic acid (L2376-500MG), and omega-6 (ω6) fatty acid, specifically *cis*-9, 12-octadecadienoic acid (L1012-100MG), were purchased from Sigma-Aldrich (St. Louis, MO, USA). Adipocyte differentiation factors, such as dexamethasone, isobutyl methylxanthine, troglitazone, insulin, platelet maturation factor human recombinant thrombopoietin (GF037), and all other molecular-biology-grade fine chemicals, were purchased from Sigma-Aldrich (St. Louis, MO, USA).

### 2.2. Cell Culture Conditions, Adipocyte Differentiation, and Platelet Maturation

Human mesenchymal stem cells (hMSCs) were cultured in DMEM supplemented with 10% FBS and 1% penicillin and streptomycin, and the cells were maintained in a humidified atmosphere containing 5% CO_2_ in an incubator at 37 °C. Early passages of hMSCs were plated in a 24-well plate at a density of 15,000 cells/cm^2^ as a monolayer and cultured with basal media. After 70% confluence of the hMSCs, adipocyte differentiation was induced by standard induction/maintenance media containing dexamethasone (1 µM), 3-isobutyl-1-methyl-xanthine (520 µM), and troglitazone (1 µM). After 72 h, the induction media was replaced with recombinant human insulin (10 µg/mL) containing maintenance media and incubated for the next 72 h [16]. Adipocyte differentiation was confirmed by the morphological observation of hMSCs transformed from a spindle-shaped fibroblastic appearance to a rounded phenotype, characterized by the accumulation of intracellular lipid droplets observed using an inverted light microscope. In addition, molecular markers like CCAAT/enhancer-binding protein alpha (C/EBPα) and peroxisome proliferator-activated receptor gamma (PPARγ) were identified in differentiated adipocytes when compared with undifferentiated hMSCs. Meanwhile, mature adipocytes were confirmed by their circular phenotype with a single large fat droplet occupying the majority of the cytoplasm, with the nucleus pushed to the periphery; molecular markers such as adiponectin and fatty acid-binding protein-4 (FABP-4) were identified in mature adipocytes when compared with early adipocytes. Differentiation-induced cells were further maintained by changing the media once every 3 days, according to the experimental design. The experiments for in vitro cell culture, adipocyte differentiation, and maintenance were carried out as per the institutional research and ethical committee guidelines of King Saud University, Riyadh, KSA.

The megakaryocytes (MEG-01) were cultured in Roswell Park Memorial Institute medium (RPMI-1640) supplemented with 10% FBS and 1% PS complex, and the cells were maintained at 37 °C in a humidified atmosphere of 5% CO_2_. For the cell maturation experiments, megakaryocytes were supplemented with 100 ng/mL of thrombopoietin (TPO) for 72 h. The cell differentiation and maturation were confirmed by an increase in size, DNA content, and the production of platelet-like particles [17].

### 2.3. In Vitro Cytotoxicity Analysis

To determine the cytotoxic effect of ω3 and ω6 against preadipocytes and hMSCs, the cells were seeded in 96-well plates at a density of 1 × 10^4^ cells/well and allowed to adhere for 24 h in growth medium (DMEM). After confirming the normal physiology of cell growth after 24 h, the medium was gently removed from the wells and replaced with fresh media containing increasing concentrations (0, 10, 20, 40, 60, 80, 100, and 120 µg/mL) of ω3 and ω6, and the cells were incubated for 24 h and 48 h, respectively. After the respective incubation period, 20 µL of MTT (5 mg/mL of PBS) was added to each well, and the wells were incubated in the dark for 4 h at 37 °C [18]. The resulting formazan crystals were centrifuged to sediment, and the supernatant was discarded carefully without disturbing the crystals. The crystals were dissolved by adding 100 μL of 100% DMSO. The absorbance was measured at 570 nm using a microplate reader (Bio-Rad, Hercules, CA, USA). Quadruplicate samples were run for each concentration of ω3 and ω6 fatty acids in three independent biological triplicates. Untreated cells were used as a negative control. The cell viability was expressed as the percentage of viable cells vs. untreated cells. The toxicity percentage was calculated using the following formula: [(Mean OD of untreated cells—Mean OD of treated cells)/(Mean OD of untreated cells)] × 100.

### 2.4. Experimental Design

To determine the intracellular lipid accumulation, cell apoptosis/necrosis, JC-1 (mitochondrial membrane potential), and adipocyte lipid metabolism-associated gene and protein profiles, the differentiated adipocytes were allowed to mature in 24-well culture plates. The respective cells were treated with different ratios of ω3/ω6 fatty acids, such as 1:1 (20:20), 1:2 (20:40), 1:3 (20:60), 1:4 (20:80), and 2:3 (40:60) (µM concentrations, with the combination not exceeding 100 µM). The cells continued to grow in a maintenance medium containing 1% insulin, and the culture media were replaced once every 72 h from ‘day 3’ to ‘day 14’ of the experimental period to confirm mature adipocytes.

**Figure 1:**
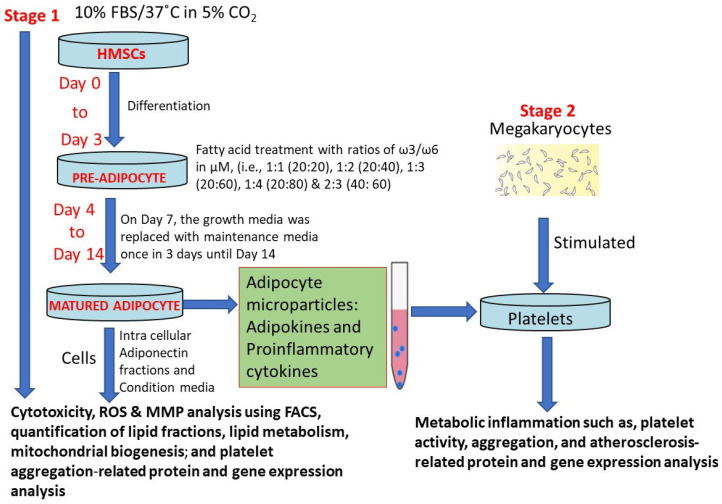
Experimental design of this study.

The conditioned media from ω3/ω6-fatty-acid-treated mature adipocytes were treated with MEG-1 (megakaryocytes) to determine the effects of adipocyte-secreted macroparticles and adipokines on the stimulation of platelet activation and aggregation. Platelets were treated with the conditioned media for 12 h, following which the gene expression patterns for platelet activation, aggregation, and metabolic inflammation were determined using reverse transcriptase–polymerase chain reaction (Figure 1).

### 2.5. Fluorescence-Activated Cell Sorting (FACS) Analysis Using Flow Cytometry

To detect the number of dead cells after 24 h and 48 h of exposure to increasing concentrations of ω3 and ω6 fatty acids, a propidium iodide/Annexin V staining assay was carried out using flow cytometry. The total cell population was pelleted using the trypsinization method, and the pellets were resuspended in 2% FBS and 0.1% sodium azide. The cells were incubated in a binding buffer with propidium iodide/Annexin V for 30 min, followed by analysis with flow cytometry (FITC Annexin V Apoptosis detection Kit-1, BD Biosciences, CA, USA).

### 2.6. Oil Red ‘O and Nile Red Staining for Lipid Accumulation Assessment

To determine the intracellular lipid accumulation, the pre-adipocytes cultured in 24-well plates were treated with different ratios of ω3 and ω6 fatty acids. The cells were maintained in a maintenance medium containing 1% insulin, and the culture media were replaced once every 72 h until day 14. According to the method of Kim et al. [19], with slight modifications, the cells were washed with PBS and stained with freshly prepared Oil Red ‘O staining solution (0.5%) for 1 h. Then, the stain was removed, and the lipid droplets were observed and photographed under a light microscope using 20× magnification. For the Nile Red staining, 5 mg of Nile Red was dissolved in 1 mL of absolute acetone. The working solution of fluorescent Nile Red stain was then prepared using 6 µL of a stock solution in 1 mL of 40% isopropanol. Then, 200 µL of the working solution was used to stain the cells for 30 min at room temperature. The stained cells were then analyzed and photographed using a fluorescence microscope.

### 2.7. Detection of Intracellular ROS by Fluorescence-Activated Cell Sorting (FACS) Analysis

The adipocyte intracellular total ROS generation level was quantified after exposure to different ratios of ω3 and ω6 fatty acids; the cells were cultured in 24-well plates and then treated with fatty acids after 24 h. In addition, pretreatment with or without 10 mM NAC for 1 h to inhibit ROS was considered a positive control. Later, the cells were washed with PBS; then, using trypsinization, cell pellets were collected. The cell pellets were resuspended with 500 µL of 10 µM DCFDA (2′,7′-dichlorodihydro-fluorescein diacetate) and incubated for 30 min. The DCFDA-treated cells were immediately processed for FACS analysis.

### 2.8. Mitochondrial Membrane Potential Assessment Using Confocal Microscopy of JC-1 Staining and Fluorescence-Activated Cell-Sorting (FACS) Analysis

The mitochondrial membrane potential was examined using the JC-1 (5,5′,6,6′-tetrachloro-1,1′,3,3′-tetraethyl benzimidazole-o-carbocyanine iodide, Sigma Aldrich) staining assay. JC-1 uptake results in green fluorescence in cells with a depolarized mitochondrial membrane, while orange fluorescence reflects cells with polarized mitochondria, converting the cationic green stain into an anionic orange color. Preadipocytes treated with ω3 and ω6 fatty acids for up to 14 days were incubated for 15 min after treatment with 5 mM of JC-1. Then, the cells were washed with JC-1 washing buffer, and the acquired signals were analyzed with a fluorescent microscope compared with untreated controls. In addition, JC-1 fluorescent staining was analyzed using the FACS method. Pretreatment with or without JC-1 was considered a positive control; a final concentration of 2.5 µM of JC-1 was added, and the cells were incubated in the dark for 15 min at 37 °C. Later, JC-1-treated cells were immediately processed for FACS analysis [20].

### 2.9. Lipid Profile Analysis

The amounts of intracellular free glycerol (lipolysis), triglycerides, free fatty acid, and lipoprotein lipase activity [21] were determined in control, untreated maturing adipocytes, and different ratios of ω3- and ω6-fatty-acid-treated adipocytes (*n* = 6) using commercial enzymatic kits (Sigma, St. Louis, MO, USA). The total protein content of the conditioned media was quantified using the Bradford method [22].

### 2.10. Reverse Transcriptase–Polymerase Chain Reaction (RT-PCR)

At the end of the experiment, total RNA was isolated, and cDNA was reverse-transcribed from the untreated mature adipocytes, different ratios of ω3- and ω6-fatty-acid-treated mature adipocytes (day 14), and mature adipocyte-conditioned media-treated platelets, using a Fastlane^®^ Cell cDNA kit (Qiagen, Hilden, Germany), according to the manufacturer’s instructions. Expression levels of adipogenesis- and lipogenesis-related genes (in adipocytes) and inflammatory genes (both in adipocytes and platelets) were quantified against the reference gene β-actin using an RT-PCR instrument (Applied Biosystem, Foster City, CA, USA). The RT-PCR was performed with a reaction volume of 25 μL, according to the manufacturer’s instructions, using gene-specific SYBR Green-based QuantiTect^®^ primers (Qiagen, Hilden, Germany). The obtained fluorescence from Ct cycles from gene targets was analyzed to determine the comparative expression level between untreated and experimental cells, respectively. The gene expression level was calculated as previously described by Yuan et al. [23]. To determine the relative expression levels, the following formula was used: ΔΔCt (comparative threshold) = ΔCt (Treated) − ΔCt (Control). Thus, the expression levels were expressed as n-fold differences relative to the reference gene. The value was used to plot the expression of genes by calculating 2^−ΔΔCt^.

### 2.11. Western Blot and Relative Quantification of Inflammatory Signaling Proteins

Protein samples were extracted from experimental cells and reacted with phospho(*p*)−prostaglandin 12-R (in adipocytes) and phospho(*p*)−thromboxane A2 (in platelets) and then quantified using the Western blot method. The antibodies for *p*−prostaglandin 12-R (APR-068-50 µL), *p*−thromboxane−a2R (APR-069-50 µL), and the secondary antibody, anti-goat-horseradish peroxidase (HRP), were obtained from Thermo Fisher Scientific company (Waltham, MA, USA). Briefly, Western blotting was performed using the extracted cell pellets, which were resuspended in protease inhibitor-containing radioimmunoprecipitation assay (RIPA) buffer (containing 50 mM Tris–HCl, with a pH of 7.4, 2 mM EDTA, 1% NP-40, 1% sodium deoxycholate, 0.1% sodium dodecyl sulfate, and 150 mM NaCl). A concentration of 10–30 μg of protein was loaded onto a 10% SDS–polyacrylamide gel for electrophoresis to sieve the proteins based on their molecular weight. The sieved proteins from the gel were transferred to a PVDF membrane, and the membranes were reacted with polyclonal anti-*p*-prostaglandin 12-R (1:5000) and anti-*p*-thromboxane-a2R (1:2000) antibody. The membranes were then incubated with horseradish peroxidase-conjugated anti-goat IgG. The internal loading control, primary-GAPDH, followed by HRP-conjugated secondary antibody, was analyzed. The levels of protein expression were determined using a chemiluminescence detection kit (ECL; Amersham Pharmacia Biotech, Bukinghamshire, UK) at various times (5, 10, and 30 min), and the scans of the shorter exposure were used for the relative quantity of optical density (OD) calculations [24]. The relative density of protein bands was quantified using the ImageJ Lab software (Image Lab Software for PC version 6.1, Bio-Rad, Hercules, CA, USA). This software interprets the band in three dimensions, with the length and width of the band density using the ‘Lane and Bands’ tool. Using the ‘quantity tool’, the relative quantity or optical density of the selected band was measured using the width of the band, which accounts for the area of peak of interest after background subtraction. The report was generated, and the relative quantity was obtained by the ‘Export results’ tool [25]. The amount of target protein was normalized to its respective GAPDH relative quantification values (detailed method presented in Supplementary File S1 Methods).

### 2.12. Statistical Analysis

The experimental data were analyzed via one-way analysis of variance (ANOVA), and statistical significance evaluation was carried out via Tukey’s multiple comparison test using the SPSS 28.5 software package [19]. The results are presented as the mean ± SD for six replications in each group [24,26], and *p*-values at the levels of *p* ≤ 0.05 and *p* ≤ 0.001 were considered to indicate significant differences between the control and treatment groups.

## 3. Results

### 3.1. Cytotoxicity

**Figure 2:**
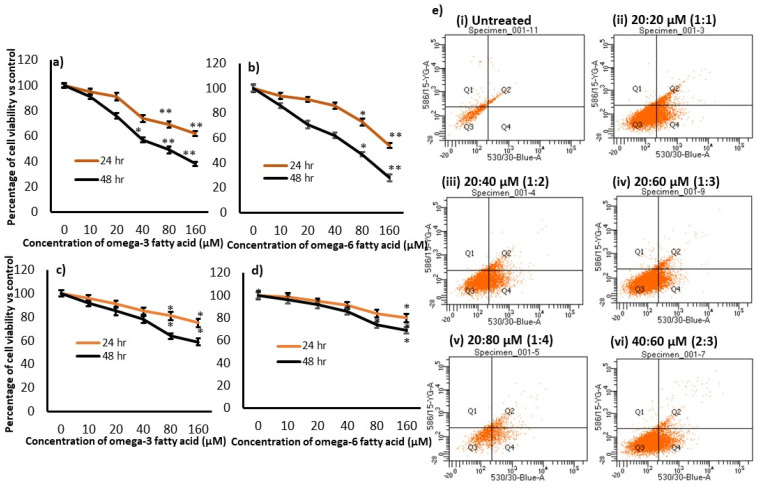
In vitro cytotoxicity assays for ω3 and ω6 fatty acids in differentiated adipocytes (**a**,**b**) and human mesenchymal stem cells (**c**,**d**), and determination of apoptosis levels at different ratios of ω3 and ω6 treatments in preadipocytes using Annexin V/PI staining after FACS (**e**). The data represent the mean ± SD of experiments performed in triplicate. In preadipocytes, the maximum inhibition of ω3 was observed as 62% (**a**), and that of ω6 was observed as 48% (**b**), after 48 h, respectively. In hMSCs, the maximum inhibition of ω3 was observed as 42% (**c**), and that of ω6 was observed as 31% (**d**), after 48 h, respectively. Figure 2e, showing FACS analysis of Annexin V/PI, indicates significantly low toxicity (4% late apoptosis (Q2) and 7% early apoptosis (Q3)) at the 1:3 (20:60 µM) ratio of ω3/ω6 when compared with all other ratios (1:1 (20:20 µM), 1:2 (20:40 µM), 1:4 (20:80 µM), and 2:3 (40:60 µM)), such as 26.9% (1:1), 35.4% (1:2), 47.8% (1:4), and 25% (2:3) of apoptosis after 48 h, respectively. In (**a**–**d**), significant values correspond to ** *p* < 0.01 and * *p* < 0.05 vs. control.

Cell viability was determined in preadipocytes and hMSCs after treatment with increasing concentrations of fatty acids. The MTT assay indicated that 10 µM and 20 µM of ω3 fatty acid were found to be safe and did not induce cell death when compared with 40 µM (23% cell death), 80 µM (36% cell death), and 160 µM (62% cell death) of ω3 fatty acid after 48 h in preadipocytes. Moreover, ω6 fatty acid concentrations of 10, 20, and 40 µM were found to be safe; however, 80 µM and 160 µM inhibited cell viability by approximately 42% and 48% after 48 h in preadipocytes (Figure 2a,b). The tested concentrations of ω3 or ω6 fatty acids in hMSCs were found to be safe; however, higher doses of ω3 at 80 and 160 µM inhibited hMSC cell viability by approximately 36% and 41%, respectively. Meanwhile, the highest dose of ω6 fatty acid at 120 µM resulted in 31% inhibition of hMSCs after 48 h (Figure 2c,d).

### 3.2. Dose Determination

Cell viability was determined in preadipocytes after treatment with different ratios of fatty acids, and the MTT assay indicated that 20, 40, 60, and 80 µM of both ω3 and ω6 did not result in significant inhibition of cell viability. Furthermore, to determine the combination and different concentrations of ω3/ω6 (µM), such as the 20:20 (1:1), 20:40 (1:2), 20:60 (1:3), 20:80 (1:4), and 40:60 (2:3) ratios of ω3/ω6 fatty acids, they were tested for their cell viability and apoptosis potential using fluorescence microscopy and Annexin V staining (the FACS method) after 48 h. Preadipocytes treated with a 1:3 ratio of ω3/ω6 fatty acids were found to have a normal cell morphology and a significantly low toxicity of 11% [4% late apoptosis (Q2) and 7% early apoptosis (Q3)] in the Annexin V FACS analysis when compared with all other treated cells. In addition, the cell viability decreased to 47.8% at the 1:4 ratio of the ω3/ω6 treatment when compared with all other treatment ratios, with cell viability inhibition observed at the 1:1 (26.9%), 1:2 (35.4%), and 1:3 (25%) ratios; late (Q2) and early (Q4) were combined for all results (Figure 2e).

### 3.3. Oil Red ‘O and Nile Red Staining for Lipid Accumulation

**Figure 3:**
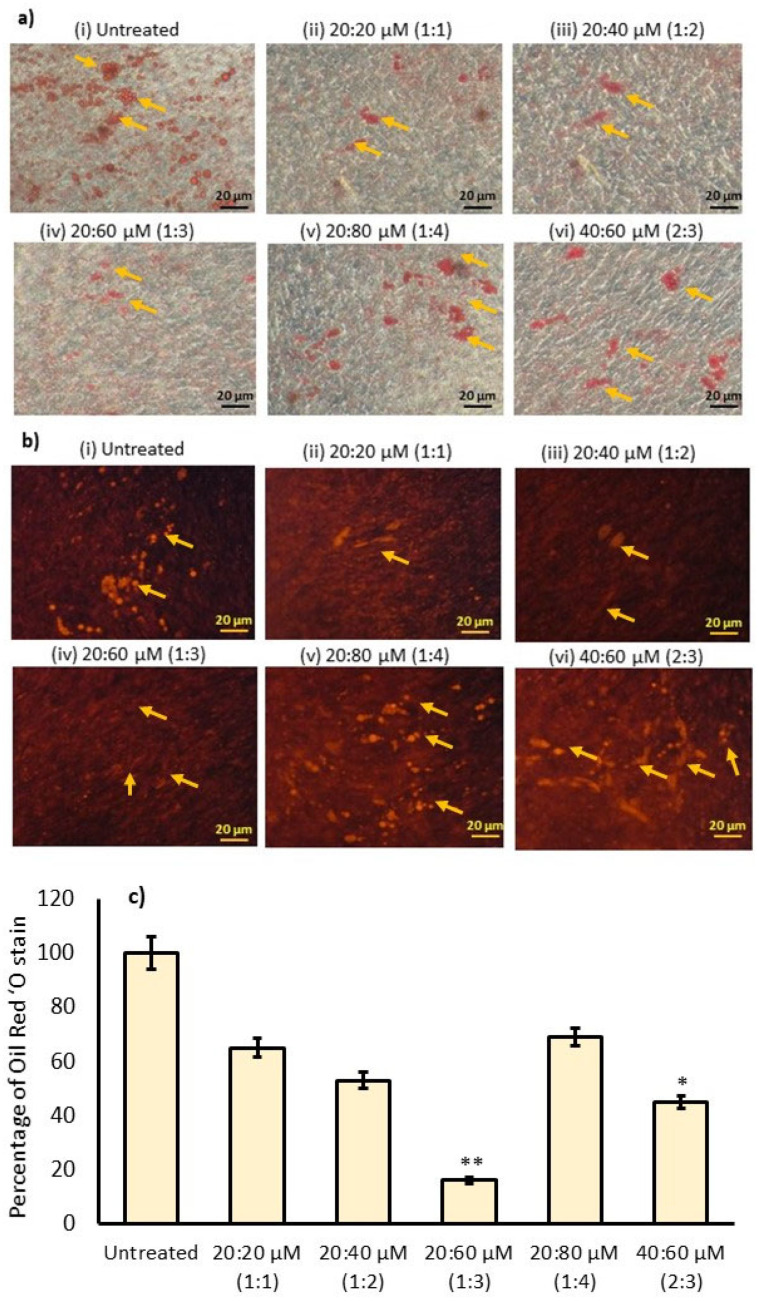
Effects of ω3 and ω6 fatty acids on inhibition of lipid accumulation in maturing adipocytes after 14 days. (**a**,**b**) Oil Red ‘O and Nile Red staining images. (**c**) The relative density of Oil Red ‘O accumulation between different ratios of ω3 and ω6 fatty acids. Data are expressed as means ± SEM. (*n* = 6). Significantly low levels of Oil Red ‘O percentages at 20:60 µM (1:3 ratio) are represented as * *p* < 0.001 against 20:80 µM (1:4 ratio) and the untreated group. ** *p* < 0.05 represents 40:60 µM (2:3 fatty acid group), showing significantly lower Oil Red ‘O percentage compared with the untreated control and 1:1 and 1:2 ratios of ω3/ω6.

**Figure 4:**
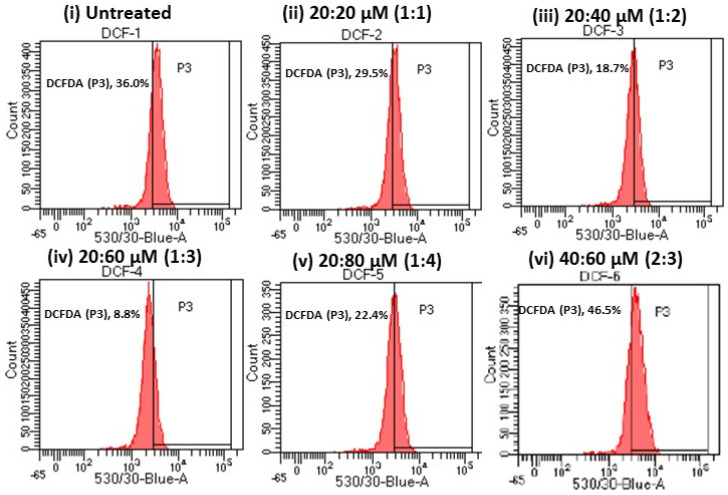
Examination of effects of different ratios of ω3 and ω6 fatty acids on ROS generation using 10 µM of a H2DCF−DA fluorescent probe for 30 min; the shift in green fluorescence intensity was determined as a percentage (P3) using FACS analysis. FACS analysis of ROS in adipocytes treated with a 20:60 μM (1:3) ratio of ω3/ω6 fatty acids showed significant decreases (18.7%) in ROS levels when compared with untreated control or other ratios of the ω3/ω6 treatment. Meanwhile, the DCF levels or ROS signal were found to be 29.5% at 1:1, 18.7% at 1:2, 22.4% at 1:4, and 46.5% at 2:4, confirming elevated levels of oxidative stress upon exposure to different ratios of ω3/ω6 fatty acids.

In Figure 3a, the 20:20, 20:40, 20:80, and 40:60 ratios of ω3/ω6-treated maturing adipocytes show significantly higher lipid accumulation with a large droplet morphology, confirmed by Oil Red ‘O staining in differentiation-induced maturing adipocytes (observed on day 14 and denoted with yellow arrowheads). However, the 20:60 ratio of ω3/ω6-treated adipocytes showed a linear shape with smaller lipid droplets in the light microscopy image analysis. Figure 4 shows a significantly decreased lipid content in the 20:60 (ω3/ω6) ratio-treated adipocytes, with an 84% reduction when compared with untreated differentiated adipocytes. Most interestingly, the observed lipid accumulation inhibitory potential of the 20:60 ratio of ω3/ω6 was significantly (*p* ≤ 0.05) higher when compared with the ratios of 20:80 (1:4) and 40:60 (2:3).

In Figure 3b, the Nile Red observation clearly discriminates the presence of lipid droplets in maturing adipocytes on day 14. At the same time, differentiated adipocytes treated with the 20:60 (1:3) ratio of ω3/ω6 showed significantly (*p* < 0.05) reduced lipid accumulation by 90% after 10 days of adipocyte maturation. The lipid accumulation inhibition observed at the other 20:40 (1:2) and 20:80 (1:4) ratios of ω3/ω6 was found to be significantly (*p* ≤ 0.001) lower when compared with the 1:3 ratio. Most interestingly, Nile Red staining clearly discriminated the WAT and BAT morphologies between untreated and different ratios of ω3/ω6-treated adipocytes. Differentiated adipocytes showed WAT behavior with larger lipid globules and a shrunken nucleus, whereas 1:3-treated maturing adipocytes showed a BAT-like physiology, with smaller oil globules surrounded by the nucleus, as shown in Figure 3b.

Quantitative measurement of Oil Red ‘O staining after extraction with isopropanol also confirmed that a ratio of 20:60 (1:3) of ω3/ω6 fatty acids showed lower absorption at 520 nm when compared with untreated adipocytes and 1:2 and 1:4 ratios of ω3/ω6-fatty-acid-treated experimental cells (Figure 3c).

### 3.4. ROS Analysis Using FACS Method

To determine the total intracellular ROS in maturing adipocytes, 2′,7′-dichlorodihydrofluorescein diacetate (DCFH-DA) staining was performed. The oxidation of DCFH-DA to 2′-7′dichlorofluorescein (DCF) has been used extensively for total ROS detection [including hydroxyl radicals (•OH) and nitrogen dioxide (•NO_2_)]. Exposure of DCFH-DA to cells allows cellular esterase to remove the acetyl groups, resulting in DCFH. Furthermore, the oxidation of DCFH by ROS converts the molecule to DCF, which emits green fluorescence at an excitation wavelength of 485 nm and an emission wavelength of 530 nm. The amount of green fluorescence (DCF) is directly proportional to the amount of total ROS levels in the cells. In FACS analysis, untreated maturing adipocytes were found to have 36% of green fluorescence (equivalent to the DCF level), but treatment with 20:60 μM or a 1:3 ratio of ω3/ω6 significantly (*p* < 0.05) reduced the DCF level to 8.8%, confirming a low level of oxidative stress. Meanwhile, the DCF levels or green fluorescence were 29.5% at 1:1, 18.7% at 1:2, 22.4% at 1:4, and 46.5% at 2:3, confirming elevated levels of oxidative stress upon exposure to different ratios of ω3/ω6 fatty acids (Figure 4).

### 3.5. Mitochondrial Membrane Potential (MMP, JC-1) Analysis

**Figure 5:**
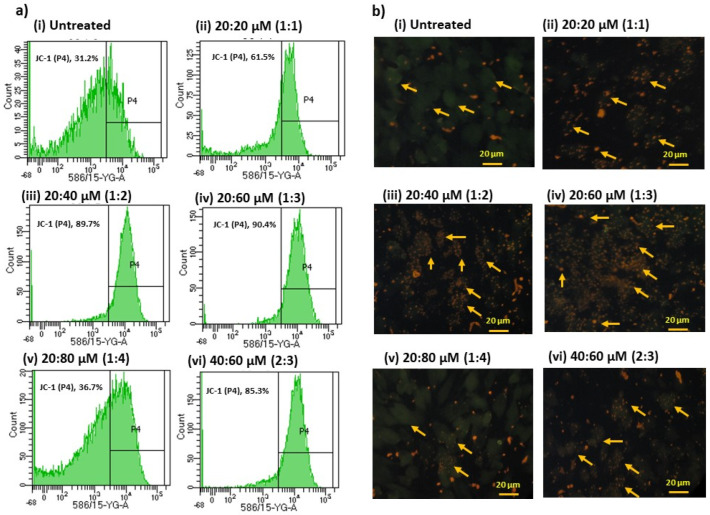
FACS and fluorescence microscopy analysis of mitochondrial membrane potential using JC-1 staining for different ratios of ω3 and ω6 fatty acids in maturing adipocytes after 14 days. (**a**) FACS analysis confirmed that the 20:60 μM (1:3) ratio of ω3/ω6-fatty-acid-treated adipocytes significantly increased (90.4%) the J-aggregate formation, indicating that a high MMP converts JC-1 monomeric dye to J-aggregate. Meanwhile, J-aggregate levels or green fluorescence were found to be 61.5% at 1:1, 89.7% at 1:2, 36.7% at 1:4, and 85.3% at 2:4, confirming the low MMP (ΔΨ_m_) at other ratios of ω3/ω6 fatty acids. (**b**) JC-1 fluorescence images showing merged images of the red and green signals of the dye (highlighted with yellow arrow heads), corresponding to JC-1 in J-aggregates vs. the monomeric form. We found fewer J-aggregates in the untreated control and the 1:4 ratio of ω3/ω6-fatty-acid-treated adipocytes. The 1:3 ratio of ω3/ω6-fatty-acid-treated adipocytes showed high J-aggregates, directly representing a high MMP (ΔΨ_m_), i.e., a high mitochondrial membrane potential, compared with all other groups.

According to the number of mitochondrial concentrations, a lipophilic fluorophore, JC-1 dye, forms J-aggregates, reflecting the mitochondrial membrane potential (Figure 5). FACS observation after JC-1 dye at the 1:3 ratio of ω3/ω6-fatty-acid-treated adipocytes significantly increased (90.4%) the J-aggregate, indicating increased JC-1 dye accumulation in the mitochondria, consistent with mitochondrial membrane polarization (Figure 5b). Meanwhile, the J-aggregate levels or green fluorescence were low at other ratios of ω3/ω6 fatty acid treatments, such as 61.5% at 1:1, 89.7% at 1:2, 36.7% at 1:4, and 85.3% at 2:3, confirming a decreased MMP (ΔΨ_m_). Figure 5b presents typical images of the JC-1 fluorescence, with each group showing merged images of the red and green colors of the dye, corresponding to JC-1 in the J-aggregate vs. the monomeric form.

### 3.6. Lipid Profile

Lipid profile analysis confirmed that adipocytes treated with a 1:3 ratio of ω3/ω6 fatty acids showed a significantly decreased amount of free fatty acids, free glycerol, and TG release, as well as lipase activity in differentiated pre-adipocytes, as shown in Table 1. Treatment with a 1:3 ratio of ω3/ω6 significantly (*p* < 0.001) reduced TG levels, free fatty acid levels, and the LPL activity of maturing adipocytes and decreased lipase activity when compared with untreated adipocytes and 1:1 and 2:3 ratios of ω3/ω6-fatty-acid-treated mature adipocytes after 14 days. Meanwhile, a 1:2 ratio of ω3/ω6 fatty acids significantly (*p* < 0.05) reduced free fatty acid and free glycerol levels, whereas a 1:4 ratio reduced free fatty acid levels and triglyceride release when compared with untreated adipocytes.

### 3.7. Effect of ω3/ω6 Fatty Acid Treatment on Gene Expression

**Figure 6:**
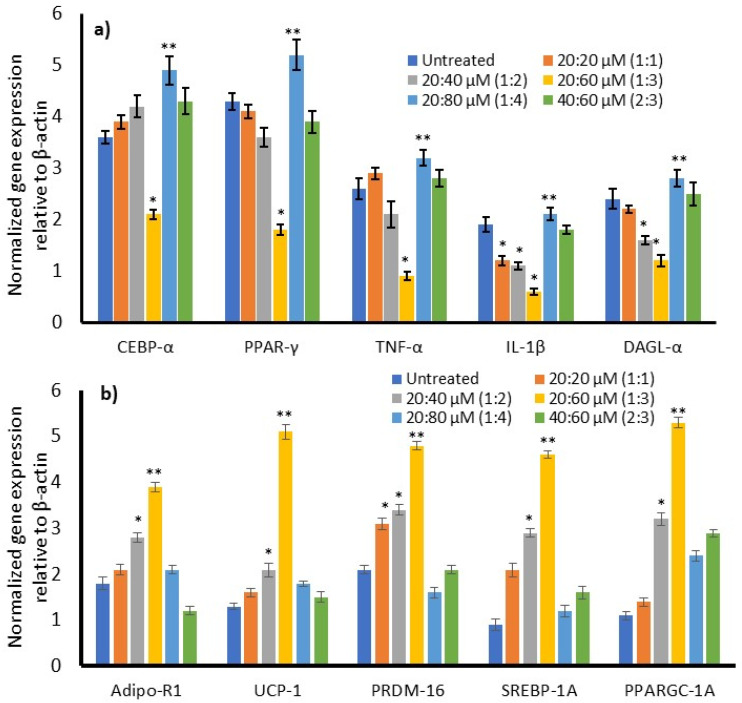
Alterations in the expression level of adipogenesis (**a**); active lipid metabolites and mitochondrial thermogenesis-related (**b**) genes in ω3- and ω6-fatty-acid-treated maturing adipocytes after 14 days. Data are expressed as means ± SEM (*n* = 6). ** *p* < 0.001 corresponding to the significant changes in gene expression at the 1:3 ratio of ω3/ω6 when compared with the 1:4 ratio group or untreated control. [*] is considered at the *p* < 0.05 level of significance at the 1:3 ratio compared with the 1:4 ratio or untreated control.

We analyzed the expression of adipokines, adipocyte lipid metabolism, fatty acid β oxidation, and oxidative mitochondrial biogenesis-related genes in maturing adipocytes after 14 days of ω3/ω6 fatty acid treatment. We found that the expression of adipogenesis-related genes, such as C/EBPα and PPARγ, and adipokines responsible for metabolic inflammation, such as TNF-α and IL-1β, were significantly (*p* ≤ 0.001) downregulated at the 1:3 ratio of ω3/ω6 fatty acid treatment after 14 days (Figure 6a). Treatment with the 2:3 ratio also increased hyperplasia and inflammatory gene expression, supporting the finding of increased ROS and suppressed MMP after 14 days. Adipocyte thermogenesis or effective utilization of fats for energy production associated with mRNAs, such as adipo-R1, UCP-1, PRDM16, PPARγC1α, and SREBP-1c, was significantly increased at the 1:3 ratio of ω3/ω6-fatty-acid-treated cells (20:60 µM) compared with untreated maturing adipocytes (Figure 6b). Most interestingly, the expressions of Adipo-R1, UCP-1, PPARγC1α, PRDM16, and SREBP-1c were upregulated two-fold higher in the 1:3 ratio group compared with the 1:4 or 1:2 ratio groups.

**Figure 7:**
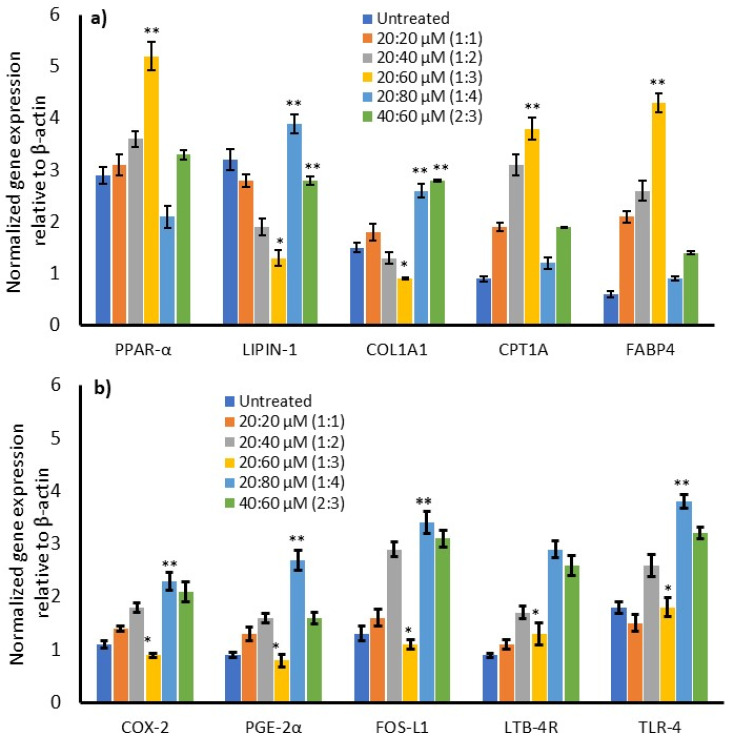
Alterations in the expression levels of adipocyte expansion, lipogenesis (**a**), and inflammatory signaling (**b**) genes in ω3- and ω6-fatty-acid-treated maturing adipocytes after 14 days. Data are expressed as means ± SEM (*n* = 6). Significant downregulation of gene expression at the 1:3 ratio of ω3/ω6 is represented as * *p* < 0.05 against the 1:4 ratio. Significant upregulation of gene expression in the 1:4 ratio group against the untreated control and the 1:3 ratio of ω3/ω6 is represented as ** *p* < 0.001.

Suppressed metabolic efficiency or thermogenesis at the 1:2 and 1:4 ratios of ω3/ω6 fatty acid treatment stimulates adipocyte hyperplasia and lipid metabolites to build an excessive cell membrane. The mRNA associated with cell expansion and endogenous signaling lipid synthesis, such as DAGL-α, LIPIN-1, COLA1, and FABP4, were significantly increased; meanwhile, PPAR-α and CPT1A expression were decreased at the 1:2 and 1:4 ratios when compared with the 1:3 ratio. The 1:3 ratio (20:60 µM) significantly decreased adipocyte hyperplasia- and lipid metabolism-stimulating mRNAs after 14 days (Figure 7a). Uncontrolled adipocyte expansion and accumulation of excessive free lipids resulted in the stimulation of inflammatory mediators, such as COX-1, PGE-2α, FOS-L1, LTB-4R, and TLR-4 levels. We found significantly upregulated inflammatory genes at the 1:1, 1:2, and 1:4 ratios of ω3/ω6-fatty-acid-treated mature adipocytes, but the condition was reversed at the 1:3 ratio after 14 days (Figure 7b).

**Figure 8:**
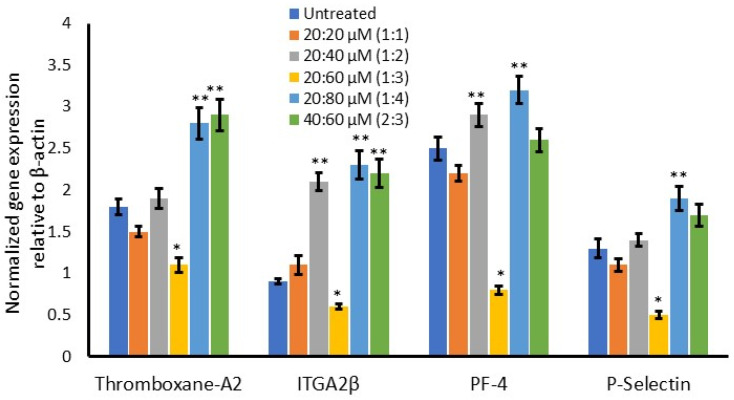
Alterations in the gene expression levels of platelet aggregation- and thrombosis-associated gene expression (fold change) in ω3- and ω6-fatty-acid-treated maturing adipocytes, with adipocyte-treated conditioned media-treated platelets after 12 h. Data are expressed as means ± SEM. (*n* = 6). Significant downregulation of inflammatory gene expression at the 1:3 ratio of ω3/ω6 is represented as * *p* < 0.05 against the 1:4 ratio group. Significant upregulation of platelet aggregation- and thrombosis-related gene expression at the 1:2, 1:4, and 2:3 ratios of ω3/ω6 against untreated control and 1:3 µM of ω3/ω6 is represented as ** *p* < 0.001.

In addition, the inflammatory gene expression level was quantified in adipocyte conditioned media-treated platelets after 12 h. We observed that platelet activation- and aggregation-related mRNA, such as thromboxane-A2, ITGA-2β, PF-4, and p-selectin expression, were significantly lowered at the 1:3 ratio of ω3/ω6-fatty-acid-treated conditioned media-treated platelets. Meanwhile, thromboxane-A2 and p-selectin expression were found to be lowered (not significantly) at the 1:1 ratio of ω3/ω6-fatty-acid-treated platelets. Most noteworthy is that the above platelet activation- and aggregation-related mRNAs were increased two-fold in the 1:4- and 2:3-ratio-treated groups when compared with the 1:3 ratio or untreated conditioned media-treated platelets (Figure 8).

### 3.8. Association Between ω3-to-ω6 Fatty Acid Ratio, Adipocyte p-Prostaglandin 12-R, and Platelet p-Thromboxane A2 Expressions

**Figure 9:**
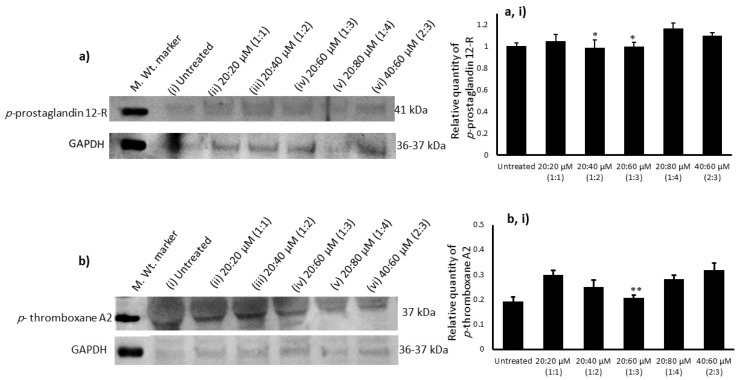
Relative quantification of protein levels of *p*-prostaglandin 12-R in ω3- and ω6-fatty-acid-treated maturing adipocytes after 14 days (**a**,**ai**), and *p*-thromboxane A2 levels in adipocyte conditioned media-treated platelets after 12 h (**b**,**bi**) in the Western blot analysis. Data are expressed as means ± SE.M. (*n* = 3). Significant values are presented as * *p* < 0.05 and ** *p* < 0.001, representing significant changes in the relative quantities of *p*-prostaglandin 12-R (adipocyte) and *p*-thromboxane A2 (platelet) at the 1:3 ratio of ω3/ω6 (20:60 µM) against the 1:4 and 2:3 ratios. In addition, the changes were not significant when compared with the 20:60 µM (1:3) and 20:20 µM (1:1) or 20:40 µM (1:2) ratios.

Adipokines and inflammatory cytokines induce pro-aggregatory mediators, which initiate platelet activation and fibrinogen formation, causing inflammation and thrombosis. The ratio of ω3 to ω6 fatty acids affects the expression of inflammatory genes in adipocytes, and secreted adipokines modulate fibrinogenic markers in platelets. We quantified the phosphorylated forms of prostaglandin 12-R (in adipocytes) and thromboxane A2 (in platelets) to detect a primary molecular switch activating inflammatory pathways upon FA treatment. The relative quantity or absolute value of *p*-prostaglandin 12-R in mature adipocytes was significantly decreased after treatment with the 1:3 ratio (20:60 µM) of ω3/ω6 fatty acids (0.997 RQ) when compared with the 1:2 (1.05 RQ) and 1:4 (1.165 RQ) ratios (Figure 9a,ai). The *p*-thromboxane A2 level was significantly lowered in platelets exposed to conditioned media from mature adipocytes treated with the 1:3 ratio of ω3/ω6 fatty acids when compared with groups exposed to all other ratios (Figure 9b). The relative quantity or absolute value of *p*-thromboxane A2 was significantly decreased at the 1:3 ratio of ω3/ω6 fatty acids (0.2066 RQ) when compared with the 1:2 (0.249 RQ) and 1:4 (0.281 RQ) ratios. The highest *p*-thromboxane A2 level in the ImageJ analysis (Image Lab Software for PC version 6.1, Bio-Rad, CA, USA) was found to be 0.317 (RQ) at the 2:3 ratio (40:60 µM) of ω3/ω6 fatty acid conditioned media-treated platelets (Figure 9bi). The above findings confirm that maintaining an optimal ω3 -o-ω6 fatty acid ratio is critical for regulating adipocyte plasticity, whereas excessive supplementation with ω3 fatty acids can adversely affect adipocyte maturation and promote platelet aggregation (Supplementary Figures S1 and S2).

## 4. Discussion

Dietary intake of highly saturated or trans fats plays a crucial role in the progression of chronic disease and fibrosis [27], while PUFAs exhibit protective effects [28,29]. PUFAs, particularly ω3 (linolenic acid, EPA, and DHA) and ω6 (linoleic acid), reduce inflammation and lower LDL-c levels, which reduce the risk of obesity, platelet activation, adipocyte fibrosis, and coronary heart disease [18]. However, to better understand the effects of an imbalance between ω3 and ω6 intake, a deeper mechanistic understanding of vascular cell remodeling is required [30]. The predominant consumption of linoleic acid (LA, 18:2) is due to its widespread availability from primary sources, such as plant oils, chickens, eggs, nuts, and meat products [31]. The daily requirement of LA is 1–2% of the total energy needed to combat essential fatty acid requirements [32,33]. In contrast, the daily consumption level of ω6 has risen to 4.9 to 21.0 g due to the increased use of vegetable oil in the Western diet, representing 4–10% of total daily calories; this increase was observed to occur within a few decades [33]. Of the PUFAs, ω3 and ω6 are desaturated and metabolized by the same set of enzymes, namely, delta-5 and delta-6 desaturase, both of which possess a higher affinity for ω3 than ω6 because they exist in a 1:4 ratio [34]. The higher availability of ω6 increases the preference of these enzymes to metabolize ω6 FAs, resulting in high quantities of arachidonic acid (AA), which can be converted into several lipoxins that determine pro- or anti-adipogenic effects, depending on the crosstalk between immune cells (M1 or M2 macrophages, neutrophils, and lymphocytes) [35,36]. Dietary surplus ω6 can be further converted into eicosanoids by the stimulation of the arachidonate–cyclooxygenase (COX) pathway and LOXs [37]. Eicosanoids are biologically active lipids and include prostaglandins (PGs), thromboxanes (TXs), and leukotrienes (LTs), collectively implicated in various activities besides inflammation, such as platelet aggregation, vasoconstriction, and fibrosis [38].

Prolonged imbalance in dietary fatty acid intake promotes excessive expansion of adipose tissue (AT), which can compromise its function and trigger systemic metabolic disturbances [39]. Beyond its role as a lipid storage organ, AT functions as an active endocrine system. Adipocytes are encircled by a specialized extracellular matrix (ECM) that not only provides structural integrity but also regulates essential cellular processes such as proliferation and differentiation [40]. Each adipocyte is sheathed in a thin pericellular ECM layer known as the basement membrane (BM), a key component separating the cells from the surrounding stroma [41]. Collagens constitute a major class of ECM proteins, among which BM-associated collagens play important roles in maintaining AT function and modulating adipocyte differentiation [42,43]. Under pathological conditions like obesity, AT frequently develops fibrosis, marked by an excess accumulation of collagen fibers that disrupts its normal architecture and function [44].

In the present study, variation in the ω3 and ω6 FA ratio was associated with altered cell viability, lipid accumulation, adipocyte morphology, and hypertrophic adipocyte features in cells treated with a higher amount of ω6 at a constant ω3 FA level. The present observations confirmed that ω3 and ω6 FAs induced cytotoxicity at doses above 40 µM and 60 µM, respectively. In this context, Smith et al. [45] also identified cytotoxicity after exposure to ω6 FA at a concentration of 100 µM in human mesenchymal stem cells. Based on these observations, the combination of *cis*,*cis*,*cis*–9,12,15–octadecatrienoic acid (ω3) to *cis*–9,12–octadecadienoic acid (ω6) was fixed within the maximum limit of 100 µM. According to these observations and previous reports, we fixed the ω3/ω6 ratios as follows: 1:1 (20:20 µM), 1:2 (20:40 µM), 1:3 (20:60 µM), 1:4 (20:80 µM), and 2:3 (40:60 µM).

A promising strategy involves boosting energy expenditure by activating brown adipose tissue (BAT) and inducing the browning of white adipose tissue (WAT) [46]. Certain dietary factors, especially ω3 polyunsaturated fatty acids (ω3 PUFAs), have attracted considerable interest for their capacity to influence adipose metabolism, suppress inflammation, and enhance thermogenesis [47,48]. While ω3 fatty acids are well known for their anti-inflammatory and lipid-modulating effects, the present study found that a 1:3 ratio of ω3 to ω6 was associated with low lipid accumulation and a lean morphology of adipocytes in the Oil Red ‘O and Nile Red analyses; however, further research is needed to clarify the mechanisms driving their role in WAT browning and BAT activation. In this context, Mazidi et al. [49] have identified that higher consumption of ω6 FA increases the risk of CVD through meta-analysis and randomized controlled trials.

Comparatively, a 1:3 ratio of ω3 to ω6 FA treatment in maturing adipocytes was associated with suppressed ROS levels, a lower percentage of apoptosis, and increased JC-1 (ΔΨ_m_) in FACS, which confirms that a favorable proportion of ω3 to ω6 FA is beneficial for adipocyte lipid metabolism via active mitochondrial biogenesis and reduced oxidative stress. Meanwhile, maturing adipocytes treated with 1:2, 1:4, and 2:3 ratios of ω3 to ω6 FA showed higher ROS levels and lower J-aggregates, corresponding to altered adipocyte metabolism and increased active lipid metabolites, such as FFA, TG, and glycerol levels, associated with the progressive initiation of oxidative stress and inflammatory signaling, as evidenced by the hypertrophic adipocyte morphology, diminished mitochondrial efficiency, accumulation of WAT, and obesity progression. In this context, Isesele et al. [50] have identified that adipocytes treated with ω6 FA-rich breast milk of non-obese women increased the levels of lipogenic genes (fatty acid synthase, acyl–CoA carboxylase, and stearoyl–CoA synthase) and that the conversion of AA and desaturase levels provided a substrate for pro-inflammatory conditions. Excessive accumulation of stearoyl–CoA desaturase (SCD–1), a catalyst for the synthesis of MUFA from saturated fatty acids in muscles, decreases fatty acid oxidation and increases TG synthesis [51]. Collectively, increased SCD1 expression following high ω6 FA intake is associated with adipocyte hyperplasia and hypertrophy [52].

Upon imbalanced dietary fatty acid intake associated with adipocyte hyperplasia and hypertrophy, stiff matrix components are deposited in the adipose tissue (AT) extracellular matrix (ECM), modifying tissue signaling lipids and the biomechanical and tensile properties of its microenvironment [53,54]. The DAGL-α (diacylglycerol lipase-α) gene regulates the diacyl glycerol lipase enzyme, which is responsible for the hydrolysis of fatty acids and the biosynthesis of endogenous signaling lipids such as DAG and arachidonic acids [55]. These consequences progressively develop following disturbances in energy expenditure and energy homeostasis, which are targets of increasing interest for obesity treatment. In the present study, a 1:3 ratio of ω3 to ω6 FA treatment in maturing adipocytes suppressed lipid signaling through reduced DAGL-α expression.

Brown adipose tissue (BAT) is recognized as a crucial site for energy expenditure through thermogenesis. It contains a high density of mitochondria, where energy from fatty acid oxidation is released as heat via the action of uncoupling protein 1 (UCP1), which is specifically expressed in brown adipocytes [56]. Activation of these brown adipocytes enhances caloric utilization and provides protection against high-fat-diet-induced adipocyte hypertrophy and hyperplasia, both of which require an elevated supply of extracellular matrix proteins, phospholipids, and sphingolipids [57]. In maturing adipocytes treated with an ω3-to-ω6 fatty acid ratio of 1:3, higher expression levels of browning markers such as UCP1, PRDM16, and SREBP1 were observed, along with reduced adipokine and inflammatory marker levels related to insulin resistance and oxidative stress. Conversely, other ω3-to-ω6 FA ratios (1:2, 1:4, and 2:3) promoted the expression of pro-inflammatory cytokines, including IL-1β and TNF-α, associated with the progression of metabolic inflammation. BAT thermogenesis, in which fatty acids derived from WAT during fasting and from the food during feeding can fully compensate for defective lipolytic machinery, plays a key role in metabolic regulation. In this context, Bay [58] reported that the susceptibility of excessive ω3 FA preparations to oxidation contributes to patient intolerance and may lead to potential toxicity and inflammation.

Obesity progression is associated with WAT fibro-inflammation, which deteriorates the insulin signaling and promotes systemic insulin resistance [59]. The prevention of adipocyte hypertrophy or excessive lipid accumulation limits adipokine levels and the demand for adipocyte cell expansion proteins, such as collagen, sphingolipid, and phospholipids [48]. Collagen and sphingolipids are the main components required for adipocyte membrane formation upon imbalance or excessive ω6 fatty acid intake. Knockdown of collagen VI limits adipocyte expansion [60]. Excessive collagen demand proportionally limits the maturation of adiponectin and increases globular adiponectin, which is associated with the pathogenesis of insulin resistance, metabolic inflammation, and the activation of platelets to synthesize fibrin for collagen biosynthesis [54,61]. In the meantime, excessive collagen deposition is linked to adipose tissue dysfunction and fibrosis in humans [62]. The LIPIN-1 and COL1A1 genes have been determined to regulate lipid metabolism and adipocyte expansion; they were downregulated in the 1:3 ratio of ω3 to ω6 fatty acids compared with the 1:2 or 1:4 ratios. This may be achieved through stimulated thermogenesis and increased expression of browning markers in 1:3-ratio-treated adipocytes.

Chronic inflammation in obesity-related diseases can lead to tissue fibrosis, a pathological outcome resulting in defects in lipid metabolites that lead to the excessive accumulation of fibrotic collagens [63]. Specifically, previous studies have raised concerns that linoleic acid (LA) and its metabolite arachidonic acid (AA) may contribute to pro-thrombotic and pro-inflammatory processes [64]. These concerns are largely based on the premise that ω6 fatty acids compete with ω3 counterparts for common enzymatic pathways, potentially limiting the synthesis of beneficial ω3-derived bioactive metabolites [65]. Evidence suggests that the lowest risk occurs when both ω6 and ω3 fatty acids are maintained at adequate levels, underscoring the importance of their balanced dietary intake. Moreover, platelets exposed to adipocyte-conditioned media demonstrated altered expression profiles of thromboxane, ITGA-2β, platelet factor-4, and p-selectin. The 1:3 ratio of ω3 to ω6 FA significantly downregulated the leukotrien-4, TLR-4, PGE-2A, FOS-L1 (Fos-related antigen–1), and COX-2 expression when compared with other ω3-to-ω6 FA ratios. Dietary practice and active lipid metabolites may serve to promote BAT thermogenesis as well as suppress fibro-inflammation through immune cell activation [66].

Our observations confirmed that a 1:3 ratio of ω3 to ω6 FA treatment in maturing adipocytes was associated with higher metabolic efficiency and significantly lower adipokine levels. The beneficial ratio of ω3 to ω6 FA has gained attention for its potential to modulate adipose tissue metabolism, reduce inflammation, and improve thermogenic capacity [67]. A promising approach is to stimulate energy expenditure by enhancing BAT activity and promoting WAT browning. Furthermore, the adipocyte-conditioned media from the 1:3 ratio of ω3 to ω6 FA significantly downregulated leukotriene-4, thromboxane-A3, MMP, and COX–2 expressions compared with other ω3-to-ω6 FA ratios in platelets. Notably, the 2:3 ratio (ω3, 40 µM: ω6, 60 µM, below the toxic level) increased oxidative inflammatory markers and platelet aggregation factors compared with the 1:3 ratio. Our findings are supported by clinical trials conducted using long-term intake of 4 g/day of ω3 supplementation instead of 1.1 g/day for females and 1.6 g/day for males [68], which was associated with a significantly increased risk of vascular cell inflammation and atrial fibrillation in individuals with established CVD or at high cardiovascular risk [30,69,70].

## 5. Conclusions

In conclusion, treatment with a 1:3 ω3/ω6 ratio (e.g., 20:60 µM) effectively promoted adipocyte differentiation into metabolically healthier, lean-type adipocytes characterized by reduced lipid accumulation, limited ECM remodeling, and attenuated inflammatory responses in platelets. These findings emphasize the significance of maintaining an optimal ω3/ω6 balance (1:3 ratio; 20:60 µM) to preserve adipocyte functionality and mitigate metabolic fibro-inflammation associated with adipose tissue dysfunction and platelet aggregation. Maintaining an optimal balance between ω3 and ω6 fatty acids is essential for the regulation of adipocyte hyperplasia and hypertrophy; meanwhile, excessive ω3 fatty acid supplementation may detrimentally influence adipocyte maturation and enhance platelet activation and aggregation. Although ω3 FAs are recognized for their anti-inflammatory and lipid-regulating properties, a better understanding of their mechanisms in promoting WAT browning and BAT activation, determination of the ideal dosage, and confirmation in in vivo studies are required.

## Figures and Tables

**Table 1:** Alterations in triglyceride, free fatty acid, and free glycerol levels, and lipase activity in ω3- and ω6-fatty-acid-treated maturing adipocytes after 14 days. Each value is expressed as the mean ± SD (*n* = 6). ^¥^—one unit of lipase is the amount of enzyme that will generate 1.0 μmole of glycerol from triglycerides per minute at 37 °C. * *p* < 0.05 compared with untreated mature adipocytes. ** *p* < 0.001 compared with untreated mature adipocytes.

Groups	Triglycerides (mg/dL)	Free Fatty Acids (mg/dL)	Free Glycerol (mg/dL)	Lipase Activity ^¥^
Untreated adipocytes	7.13 ± 0.32	4.26 ± 0.31	3.24 ± 0.31	0.24 ± 0.03
20:20 µM (1:1)	6.37 ± 0.24	4.17 ± 0.29	2.63 ± 0.23	0.16 ± 0.04
20:40 µM (1:2)	6.01 ± 0.41	2.96 ± 0.18 *	2.51 ± 0.19 *	0.14 ± 0.02 *
20:60 µM (1:3)	3.09 ± 0.33 **	1.06 ± 0.09 **	2.01 ± 0.11 **	0.06 ± 0.02 **
20:80 µM (1:4)	5.93 ± 0.47 *	2.81 ± 0.14 *	2.95 ± 0.17	0.19 ± 0.06
40:60 µM (2:3)	4.91 ± 0.52	3.93 ± 0.19	3.21 ± 0.26	0.21 ± 0.09

## Data Availability

The raw data supporting the conclusions of this article will be made available by the authors upon request.

## Supplementary Materials

The following supporting information can be downloaded at https://www.mdpi.com/article/10.3390/metabo16040271/s1: Supplementary Figure S1. Uncropped gel image for *p*-prostaglandin 12-R (41 kDa) and GAPDH in ω3- and ω6-fatty-acid-treated maturing adipocytes after 14 days. Supplementary Figure S2. Uncropped gel image for *p*-thromboxane A2 (37 kDa) and GAPDH level in adipocyte-conditioned media-treated platelets after 12 h in Western blot analysis [24,25] (Supplementary Materials are presented with methods and references).

## Abbreviations

AA	Arachidonic acid
AT	Adipose tissue
ATCC	American Type Culture Collection
BAT	Brown adipose tissue
COL1A1	Collagen type i alpha 1 chain
DAGL-α	Diacylglycerol lipase-α
DHA	Docosahexaenoic acid
EPA	Eicosapentaenoic acid
ECM	Extracellular matrix
FACS	Fluorescence-activated cell sorting
FOS-L1	Fos-related antigen-1
LTs	Leukotrienes
LA	Linoleic acid
MMPs	Matrix metalloproteases
MAGL	Monoacylglycerol lipase
PUFAs	Polyunsaturated fatty acids
SCD-1	Stearoyl-CoA desaturase
TXs	Thromboxane
UCP1	Uncoupling protein 1
WAT	White adipose tissue
