# Supplementary material for: Dynamic Role of Omega-3/Omega-6 Polyunsaturated Fatty Acid Ratio in Modulation of Adipogenicity, Lipid Metabolites, and Adipokines Associated with Platelet Hyperactivity
Source: Metabolites. 2026 Apr 17;16(4):271. doi: 10.3390/metabo16040271 (PMC13118186; doi:10.3390/metabo16040271)
Supplement: Supplementary file 1 [file metabolites-16-00271-s001.zip › metabolites-4213227-supplementary.pdf]

Supplementary materials

S1. Methods

S1.1. Western Blot and Relative Quantification of Inflammtory Signaling proteins

Protein samples were extracted from experimental cells, reacted with phospho(*p*)–prostaglandin 12-R (in adipocyte) and phospho(*p*)–thromboxane A2 (in platelets), and quantified using the Western blot method. The antibodies for *p*-prostaglandin 12-R (APR-068-50μL), *p*-thromboxane-a2R (APR-069-50μL), and secondary antibody, anti-goat-horseradish peroxidase (HRP), were obtained from Thermo Fisher Scientific company (Waltham, MA, USA). Briefly, Western blotting was performed using the extracted cell pellets from the experimental cells, which were resuspended in protease inhibitor-containing radioimmuno precipitation assay (RIPA) buffer (containing 50 mM Tris–HCl, with a pH of 7.4, 2 mM EDTA, 1% NP-40, 1% sodium deoxycholate, 0.1% sodium dodecyl sulfate, and 150 mM NaCl). A total protein concentration of 10–30 μg was subjected to 10% SDS-polyacrylamide gel electrophoresis to separate proteins based on their molecular weight. The separated proteins from gel were transferred to a PVDF membrane, and the proteins were reacted with polyclonal anti-*p*-prostaglandin 12-R (1:5000) and anti-*p*-thromboxane-a2R (1:2000) antibodies. The membrane was then incubated with horseradish peroxidase-conjugated anti-goat IgG. The internal loading control, primary-GAPDH, followed by HRP-conjugated secondary antibody, was analyzed. The levels of protein expression were determined using a chemiluminescence detection kit (ECL; Amersham Pharmacia Biotech) at various times (5, 10, and 30 minutes), and scans with shorter exposures were used for relative optical density (OD) calculations [24]. The relative density of protein band was quantified using the ImageJ Lab software (Image Lab Software for PC version 6.1, Bio-Rad, CA, USA).

S1.2. *Relative quantification using ImageJ Lab software*

The Image Lab Software for PC (Version 6.1, Bio-Rad) was employed for the acquisition and densitometric quantification of blot images. This software processes raw image data in three dimensions, where the band's length and width are specified using the "Lanes and Bands" and "Lane Profile" tools, while the emitted chemiluminescent signal appears as a vertical peak above the blot surface. The intensity of each band was determined by calculating the total volume beneath this three-dimensional peak, which can also be represented in two dimensions through the "Lane Profile" view to fine-tune the band width and accurately define the area under the curve. Background correction was performed using the "Lanes" tool with the rolling disk option. The rolling disk parameters were selected to ensure

consistent background subtraction across lanes. Using ‘quantity tool’, the relative quantity or optical density of the selected band was measured using the width of the band, which accounts for the area of peak of interest after background subtraction. The report was generated, and the relative quantity was obtained using the ‘Export results’ tool [25].

S2. Supplementary figures

Supplementary figure S1. Uncropped gel image for *p*-prostaglandin 12-R (41kDa) in omega-3 (ω3)- and omega-6 (ω6)-fatty acid treated maturing adipocytes after 14 days.

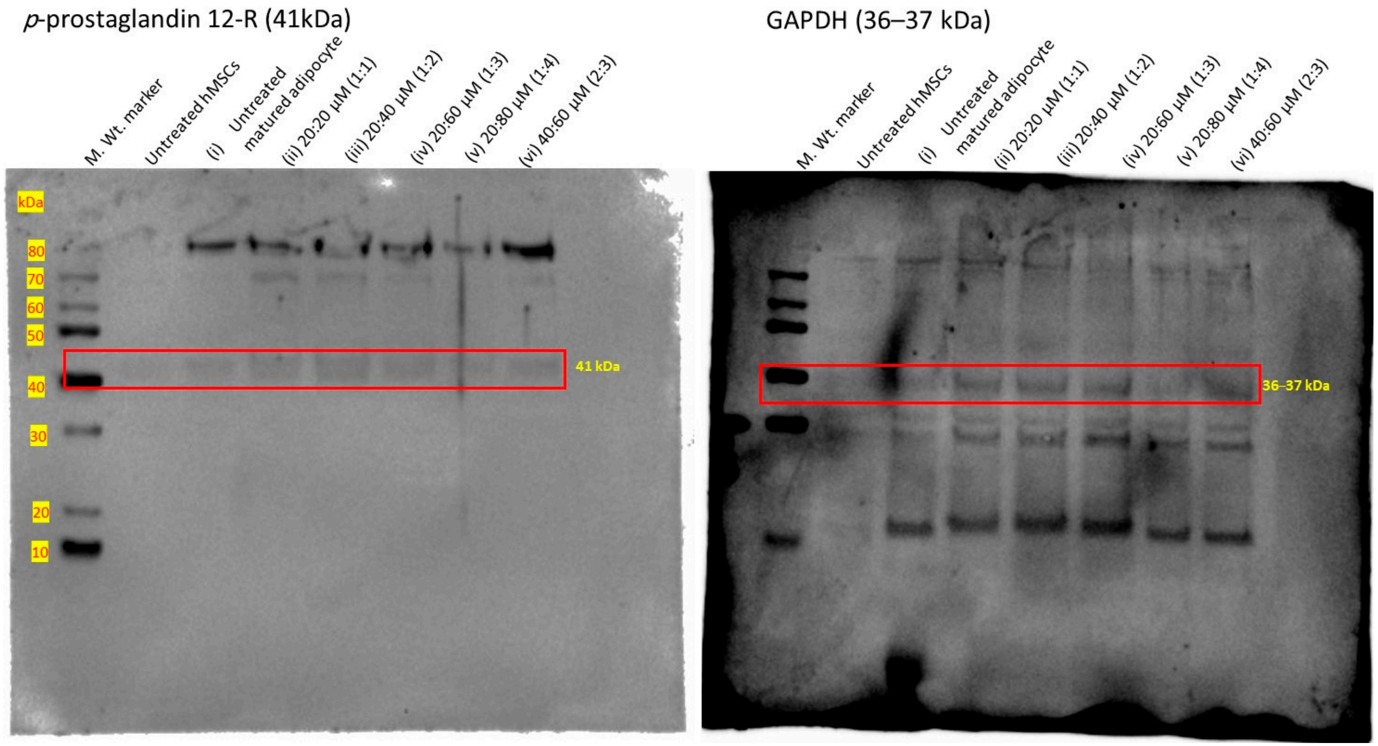

Supplementary figure S2. Uncropped gel image for phospho-thromboxane A2 (37 kDa) level in adipocyte-conditioned media-treated platelets after 12 hr in Western blot analysis.

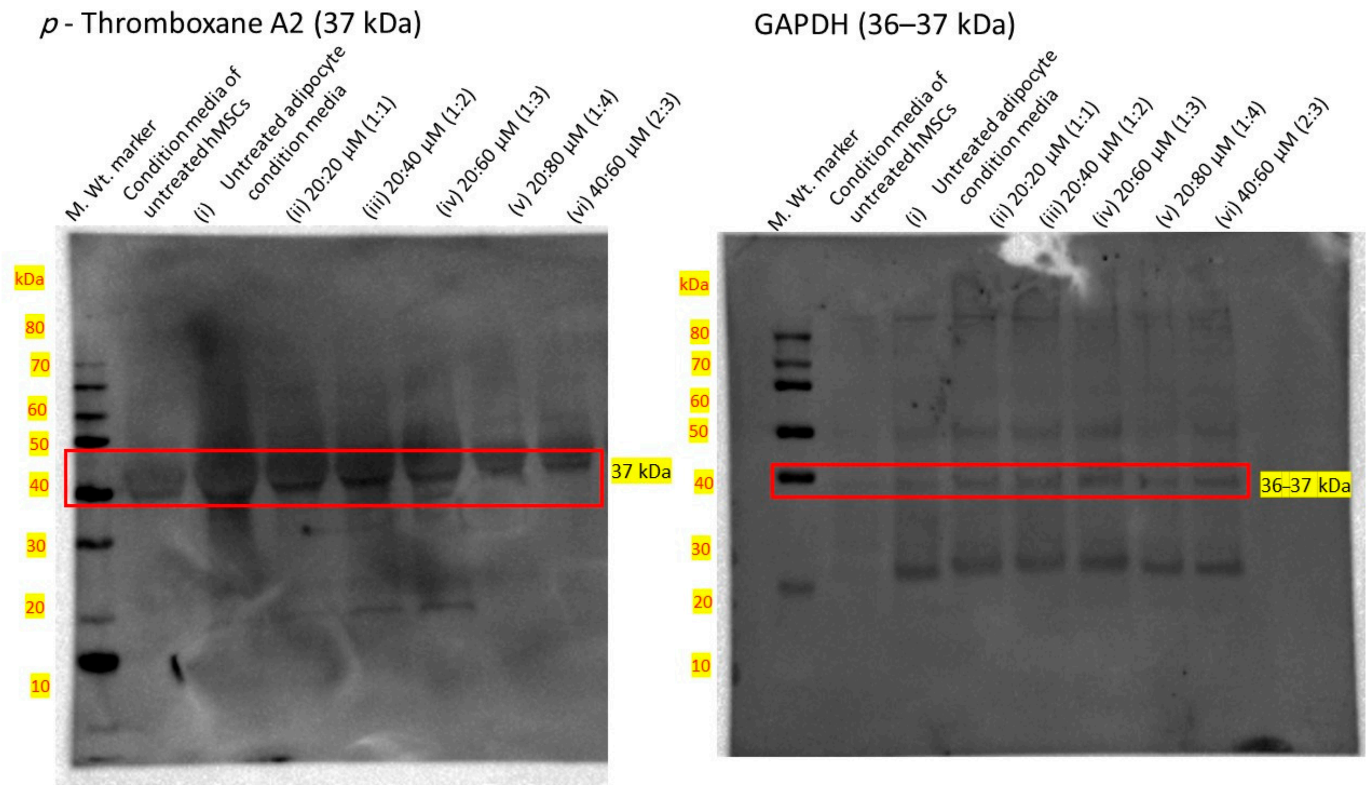

Supplementary figure S1. The uncropped blot with a molecular weight marker is presented in Supplementary Figures S1 and S2. In Figure S1, the second lane has been updated from hMSCs to untreated hMSCs; the third lane has been updated as untreated mature adipocytes. In Figure S2, the second lane has been updated from hMSCs to conditioned media of hMSCs.

S.3. Results

Quantification of phospho-prostaglandin 12-R blot data is presented only for untreated and five different ratios of ω3/ω6-fatty-acid-treated mature adipocytes. Protein quantification data for thromboxane A2 level in platelets after 12 hr is presented only for untreated adipocyte conditioned media and five different ratios of ω3/ω6-fatty-acid-treated conditioned media-treated platelets. In the ImageJ analysis, while processing the calibrate command manually, the lanes for the molecular weight marker, untreated hMSCs, and conditioned media of hMSCs were not marked or selected during band detection. The remaining target/treatment bands are presented as relative quantity values in Supplementary Table ST1.
